# Complete genome sequence of *Pediococcus pentosaceus* TOKAI 10m isolated from Japanese Takuan pickle

**DOI:** 10.1128/mra.00738-25

**Published:** 2025-11-12

**Authors:** Tomoyuki Hibi, Yuki Nakashima, Shin Yasuda, Masashi Hirano, Hideki Kinoshita, Takahiro Kawanabe

**Affiliations:** 1 Graduate School of Bioscience, Tokai University 208559, Kumamoto, Japan; 2 Research Institute of Agriculture, Tokai University 208559, Kumamoto, Japan; 3 Undergraduate School of Agriculture, Tokai University 208559, Kumamoto, Japan; 4 JSPS Research Fellowship for Young Scientists, Tokai University 208559, Kumamoto, Japan; 5 Probio Co. Ltd, Kumamoto, Japan; Fluxus Inc., Sunnyvale, California, USA

**Keywords:** complete genome, lactic acid bacteria, *Pediococcus pentosaceus*, Illumina, Oxford Nanopore Technologies

## Abstract

*Pediococcus pentosaceus* TOKAI 10 m, isolated from Japanese Takuan pickle, shows potential as a soy milk yogurt starter. Its genome comprises a 1,788,781 bp chromosome and four plasmids (56,333, 13,649, 10,458, and 10,048 bp), with no virulence factors or antibiotic resistance genes detected.

## ANNOUNCEMENT

*Pediococcus pentosaceus* TOKAI 10 m (TK10m), initially identified as *Lactobacillus sakei* MYU 10 (1) and later reclassified and renamed TOKAI 10 m (2). Briefly, 1 g of finely chopped Japanese homemade Takuan pickle was suspended in 10 mL of distilled water, and 0.1 mL of aliquot was plated on De Man, Rogosa, and Sharpe (MRS) (Difco Laboratories, USA) agar and cultured anaerobically at 37°C for 2 days using AnaeroPackTM Anaero (Mitsubishi Gas Chemical, Japan). A single colony was inoculated in MRS broth and cultured aerobically at 37°C for 24 h (1). The 16S rDNA analysis was performed using the 27 f (5′-AGAGTTTGATCCTGGCTCAG-3′) and 1525 r (5′-AGAAAGGAGGTGATCCAGCC-3′) (3). The homology search using BLAST (https://blast.ncbi.nlm.nih.gov/Blast.cgi) showed high identities (99.32%) with *P. pentosaceus* padder1 (PV916721.1) (2). TK10m can coagulate soy milk (4) and exhibits heavy metal biosorption (1) and functional small RNA production (2). However, since the genes related to the safety and functionality of this strain remained unknown, we performed whole-genome sequencing.

For short-read sequencing, TK10m was cultivated aerobically in 40 mL of MRS broth (Difco Laboratories) at 37°C overnight and harvested via centrifugation. Genomic DNA was extracted using the DNeasy Blood & Tissue Kit (QIAGEN, Germany), and its quality and concentration were assessed using Qubit (Thermo Fisher Scientific, USA). Libraries were prepared using the MiSeq Reagent Kit v3 (Illumina, USA) and sequenced on the Illumina MiSeq platform (PE300). Sequencing produced 2,902,522 reads, totaling 511,484,556 bp. Raw reads were trimmed using fastp v0.24.0 (5) (6) with the parameters “-q 30 -n 20 -t 1 -T 1”.

For long-read sequencing, TK10m was cultured in 5 mL of MRS broth under identical conditions. Genomic DNA was extracted using the Wizard Genomic DNA Purification Kit (Promega, USA). Quality and concentration were evaluated using NanoDrop 2000c (Thermo Fisher Scientific) and Quantus (Promega). Libraries were prepared using the Rapid Sequencing Kit V14 (SQK-RAD114, Oxford Nanopore Technologies [ONT], UK) without size selection and sequenced on a MinION Mk1B (ONT) employing an R10.4.1 Flow Cell (FLO-FLG114, ONT). Base calling was conducted using Dorado v7.6.8 (high-accuracy model v4.3.0, 400 bps) (https://github.com/nanoporetech/dorado). Long-read quality was assessed using NanoPlot v1.44.1 (7), generating 63,812 reads with an N50 of 18,997 bp. Reads were trimmed using NanoFilt v2.8.0 (8) with the parameters “-q 10 --headcrop 50 -l 1000”.

Hybrid genome assembly was performed using Unicycler v0.5.1 (9), and the assembly graph was visualized using Bandage v0.8.1 (10). Trimmed short reads were mapped using BWA-MEM2 v2.2.1 (11), indexed using SAMtools v1.21 (12), and employed for polishing with Pilon v1.24 (13). Genome completeness, taxonomy, and annotation were determined using DFAST v1.3.6 (14), with the chromosome rotated to initiate at the *dnaA* gene. Genome coverage was calculated by mapping trimmed reads using minimap2 v2.29 (15) (16) and SAMtools v1.21 (12). Default parameters were used for all software unless otherwise noted. Annotated genomic features are summarized in Table 1.

**TABLE 1:** Accession numbers and sequencing statistics

Bacterial species	*Pediococcus pentosaceus*
Strain	TOKAI 10 m

Assembly statistics	
Total length (bp)	1,879,269
Chromosome length (bp)	1,788,781
Plasmid 1 length (bp)	56,333
Plasmid 2 length (bp)	13,649
Plasmid 3 length (bp)	10,458
Plasmid 4 length (bp)	10,048
No. of contigs	5
Chromosome mean depth (×)	383.353
Plasmid 1 mean depth (×)	250.608
Plasmid 2 mean depth (×)	1,242.73
Plasmid 3 mean depth (×)	477.87
Plasmid 4 mean depth (×)	629.244
GC content (%)	37.2
No. of CDSs	1,918
No. of rRNAs	15
No. of tRNAs	55
No. of CRISPRS	1
Completeness check (%)	91.49
Taxonomy Check (%) (ANI to *Pediococcus pentosaceus* NBRC 107768 [GCA_007992275.1])	98.7

Finally, virulence and antibiotic resistance genes were screened using PathogenFinder v1.1 (17) and AMRFinderPlus v4.0.23 (database version: 2025-07-16.1) (18) with the parameter “-i 0.6.” However, no virulence or antibiotic resistance genes were detected in this study.

## Data Availability

Data availability. All datasetsdata sets have been deposited in DDBJ/ENA/GenBank, and the accession numbers are listed below. BioProject: PRJDB35427 BioSample: SAMD01038017 Raw short reads data: DRR698281 Raw long-reads data: DRR698282 Assembled and annotated reads data: AP041085, AP041086, AP041087, AP041088, and AP041089.
